# Supplementary material for: Patterns of Intron Gain and Loss in Fungi
Source: PLoS Biol. 2004 Nov 30;2(12):e422. doi: 10.1371/journal.pbio.0020422 (PMC532390; doi:10.1371/journal.pbio.0020422)
Supplement: Table S1 — Also available at http://genes.mit.edu/NielsenEtAl/. (4.3 MB ZIP). [file pbio.0020422.st001.zip › NielsenEtAl/html/1389.html]

AN0924.1.NCU00490.1.MG05674.1.FG05434.1


```
 CLUSTAL W (1.82) Multiple Sequence Alignments - Introns Inserted


Sequence 1: NCU00490.1	375 aa
Sequence 2: MG05674.1	437 aa
Sequence 3: FG05434.1	404 aa
Sequence 4: AN0924.1	441 aa
Alignment Length: 506 aa
Number Identitical Residues: 197 aa
Alignment Score (without introns) 9268


MG05674.1 	--------------------MMLEE---K~YIGLALAITSSLAIG1TTSDLGAN~PSDEH
NCU00490.1	-----------------------------~-------MTSSLAI-~--------~-----
FG05434.1 	---------------------MIED---K2YIGLALAMTSALAIG1TSFVITKK0G----
AN0924.1  	MISDKYASPRSLLLPVLGGRILTDDSINR~YIGLLLAILSTMAIG1TSFVITKI0G----
          	  :.. ::. :   .  ..    ..: .    .   :: *::**. ::            

MG05674.1 	VLRRLHLMASCHVQKVDNYFAQGLQQAEERLGFEGDGYVYLKNPLWWAGIG---------
NCU00490.1	------------------------------------------------------------
FG05434.1 	-----------------------LIQAEERHGFEGDGFVYLKNPLWWAGIA---------
AN0924.1  	-----------------------LNHATERHGFEGEGFSYLKSPTWWVGVSTCTEQPHLK
          	                          : .  . ....    ...    . .:.:...  .

MG05674.1 	---------TL1VLGEICNFAAYAFAPAILVTPLGALSVLIG~AILGSYFLNELLGILGK
NCU00490.1	----------V1VAGEICNFAAYAFAPAILVTPLGALAVLIG~AVLGSYFLNEELGTLGK
FG05434.1 	---------TL1ALGEICNFAAYAFAPAILVTPLGALSVLIG~AVLGSYFLKEELGTLGK
AN0924.1  	NREGEADNFAV~AIGEAANFAAYAFAPAILVTPLGALSVLIG2AVLGSYFLKERLGVLGK
          	. ...:.. :: . ** .*******************:**** *:******:* ** ***

MG05674.1 	LGSAICLIGAVIIVLHAPPDKEISTIDEILNYAIQPG1FLLYVFFVTVYATFMIYWVAPK
NCU00490.1	LGSAICLIGAVIIVLHAPPDEDIQTIDQILNYAIQPG1FLLYSALVCGFAGFMIYRVAPV
FG05434.1 	LGSAICLIGAVIIVLHAPPDEEIETVDEILHLAIQPG1FLLYAFAVVGFAVFMIYRIAPR
AN0924.1  	LGCAMCLLGSVVIVLHAPPDQPVDTIDEILQYALQPG1FLIYCLAVAIFATVMIYRVAPI
          	**.*:**:*:*:********: :.*:*:**: *:*** **:*   *  :* .*** :** 

MG05674.1 	HGKKNPLIYLSICSLVGSVSVMSVKAFGIALKLTFAGNNQFSHPSTYVFMIITVVCILIQ
NCU00490.1	YGKKNPLVYLSICSTVGSISVMAVKAFGIALKLTFAGNNQFTHPSTYVFMIITAVCILTQ
FG05434.1 	YGKKNALIYLSICSTVGSISVMSVKAFGIALKLTFAGHNQFSHPSTYVFMILTAVCILTQ
AN0924.1  	YGKRNPLIYISICSTVGSVSVMSVKAFGIALKLTLGGHNQFTHASTYVFLIVTAFCILTQ
          	:**:*.*:*:**** ***:***:***********:.*:***:*.*****:*:*..*** *

MG05674.1 	MNYFNKALSQFPTNI2VNPLYYVTFTTATLCASFILFSGFNTTDPVGTLSLLCGFLVTFT
NCU00490.1	MNYFNKALSQFPTNI2VNPLYYVTFTTATLCASFILFKGFNTTEPVATLSLLCGFLVTFT
FG05434.1 	MNYFNKALANFPTNI2VNPLYYVTFTTATLCASFILFTGFNTNDPVNTLSLLCGFLVTFT
AN0924.1  	MNYFNKALNEFSTSI2VNPLYYVTFTTATLCASFILFKGFNTTDAVNTISLLCGFLIIFS
          	******** :*.*.* *********************.****.:.* *:*******: *:

MG05674.1 	GVYLLNLSRTDPSGMR-LANGQRGDDATGTDMVSSIQTRLSMEARRSLSHRRMSAGSG--
NCU00490.1	GVYLLNLSRGDPDGTKHLARRSRGD-ATGTDMISGLQTRMSMERRRSLG--RMSTGS---
FG05434.1 	GVYLLNLSRGDPHGQR-LSAGRGGSDATGTDMVSGLQTRLSMQARRSGDPSRHSISS---
AN0924.1  	GVYLLNISRNDPDGHS--MNAKYDDEGVPTDGIAGFQTRRSMQARRSGEPHRRSSSSIAF
          	******:** ** *         ..... ** ::.:*** **: ***    * * .* : 

MG05674.1 	-THAADRQGLMRAYDEEEAAGLSLGDLADDSDEGS--------GSGSASNGKKMNG----
NCU00490.1	---NRDRDSLMRAYDEEEALG--LADLAEDSEEEDDDPRSPMIGSFRHSNGGPVNGNTTT
FG05434.1 	--QRGDREGLIRAYDEEEAAGFALTDLADDSDDSD--------DDLRRTNG---NG----
AN0924.1  	LSGRGDREGLIRSYDVE-NQNFGLSELTEESDGEPG-------PTFKRSEE---------
          	 :   **:.*:*:** *   . .* :*:::*:   .            ::          

MG05674.1 	-----------NGEPKRYDASIELQTRRQP-ER
NCU00490.1	TSTTQGGRLAPNGKKHAVGDSIEMQNRKKSGER
FG05434.1 	-----------NGKAT-YDNSIELDNRHKSGDR
AN0924.1  	-------------------VDRTSQSRKKNDDL
          	                    .   :.*:: .:
```
